# Empowering Change: A Systematic Review of the Challenges Faced by Female Healthcare Workers in Pakistan

**DOI:** 10.7759/cureus.107541

**Published:** 2026-04-22

**Authors:** Maryam Afzal, Diana Turuzhbaeva, Hala Rahman, Ajay Kausheic Madhusuthanan, Seemab Mahmood, Iana A Malasevskaia

**Affiliations:** 1 Dentistry, Rural Health Centre, Rawalpindi, PAK; 2 Internal Medicine, Samara State Medical University, Samara, RUS; 3 Plastic and Reconstructive Surgery, Manchester University NHS Foundation Trust, Manchester, GBR; 4 Emergency Medicine, Apollo Hospitals, Chennai, IND; 5 Dentistry, Combined Military Hospital (CMH) Lahore Medical College and Institute of Dentistry, Lahore, PAK; 6 Hospital-Based Medicine, Harvard T.H. Chan School of Public Health, Boston, USA

**Keywords:** female healthcare workers, gender disparity, pakistan, socio-cultural challenges, systematic review, workplace challenges

## Abstract

Pakistan's female healthcare force faces numerous challenges compared to their male counterparts that hinder their professional development. These systemic barriers contribute to mental health struggles and high attrition rates among women in the healthcare sector. This systematic review aims to identify and analyse the major difficulties encountered by female healthcare workers in Pakistan, focusing on socio-cultural and institutional factors, and to propose strategies for empowerment.

The review was conducted following the Preferred Reporting Items for Systematic Reviews and Meta-Analyses (PRISMA) 2020 guidelines. A comprehensive literature search was performed across multiple databases, including PubMed/Medical Literature Analysis and Retrieval System Online (MEDLINE), Cochrane Library, Europe PubMed Central (Europe PMC), ScienceDirect, Elton B. Stephens Company (EBSCO) Open Dissertations, and ClinicalTrials.gov, from November 22 to December 5, 2024. Ultimately, 14 studies met the inclusion criteria, focusing on workplace and socio-cultural challenges faced by female healthcare workers. Quality appraisal was conducted using the Critical Appraisal Skills Programme (CASP) checklist for qualitative studies and the Joanna Briggs Institute (JBI) critical appraisal checklist for analytical cross-sectional studies.

The findings highlight significant barriers such as workplace discrimination and social and cultural expectations that impede the professional growth of female healthcare workers. Women from lower socio-economic backgrounds face compounded obstacles, further entrenching gender disparities. The review also identifies a notable gap in female representation in leadership roles, underscoring the need for targeted interventions, including mentorship programs.

Of 1,161 records identified, 14 studies met the inclusion criteria, comprising nine qualitative studies (64.3%) and five cross-sectional studies (35.7%). Professional barriers (workplace discrimination, harassment, lack of mentorship) were reported in 13 studies (92.9%), cultural impediments in five studies (35.7%), familial constraints in seven studies (50%), work-life balance issues in five studies (35.7%), and safety concerns in seven studies (50%). Women from lower socio-economic backgrounds faced compounded obstacles. A notable gap in female representation in leadership roles was identified across multiple studies.

Addressing the multifaceted challenges endured by female healthcare workers in Pakistan requires a comprehensive approach that includes policy changes and organisational support. Implementing targeted interventions can foster an inclusive environment that empowers female healthcare professionals, ultimately enhancing the quality of care in Pakistan.

## Introduction and background

Retaining healthcare workers in low- and middle-income countries (LMICs) is a challenge, as they already face a shortage of 18 million healthcare employees [1]. Both male and female counterparts are essential to a sound healthcare system. However, globally, gender disparity is observed in the health professions, with only 26% of females holding representative positions [2,3]. The issues persist globally, not just in LMICs, as women in Japan, Saudi Arabia, and the Middle East also face a setback due to culture belief system. Similarly, female healthcare workers also struggle to have fairness in the United Kingdom [4].

Pakistan's healthcare sector is struggling with numerous issues as well, such as underfunding, a shortage of trained staff, insufficient medicines, inadequate equipment, brain drain among doctors, and significant gender-based discrimination, coupled with the underrepresentation of female staff in the healthcare workforce [5,6]. According to the World Economic Forum's gender disparity report released on June 11, 2025, sadly, Pakistan ranked last among 148 countries, highlighting the deep structural inequalities that permeate all sectors, including healthcare [7].

The system in terms of leadership roles is predominantly male-dominated, and women in Pakistan's healthcare system confront particular challenges, including discrimination, harassment, and work-life balance concerns, which negatively influence their professional advancement and mental well-being [8,9].

As per existing literature, the Pakistan Medical and Dental Council has 383,568 doctors and dentists registered with them, including 171,482 female doctors, now slightly outnumbering their male counterparts (168,577). However, nearly 35% (approximately 60,019 of 171,482) of women doctors remain unemployed, with work-family conflict affecting 37-58% of female physicians. For many, marriage becomes a barrier, as spouses and in-laws often discourage clinical practice, leading to early career attrition. Each medical graduate represents a substantial public investment. Losing even a single graduate leads to significant financial waste, along with the individual's lost time, effort, and potential [10]. The nursing profession has a higher proportion of women, around 90%. Yet, overwhelming working hours, pressure, intimidation by perpetrators, and reduced pay scales lead to about one-third resigning, within the first two years of their employment, resulting in a shortage of more than 60,000 nurses in Pakistan [11,12]. Additionally, the militant situation in provinces like Khyber Pakhtunkhwa has jeopardised the lives of many lady health workers [13].

Female physicians also have limited opportunities for professional advancement in academic and medical settings. A recent qualitative study exploring the experiences of Pakistani women in healthcare leadership positions revealed that women face significant barriers including unsupportive work environments, gender-based stereotyping, male-dominated hierarchies, and resistance to female authority. The study identified that most of these obstacles stem from deeply entrenched patriarchal norms that assign conventional gender roles, making it difficult for women to acquire and sustain leadership positions despite their professional competence [14]. Inadequate hiring procedures, stringent transfer regulations, and persistent harassment at work are commonplace for female physicians. This significantly inhibits women's access to senior positions while maintaining long-standing disparities in promotion and decision [10].

This situation is deeply concerning. The declining availability of female doctors, especially in conservative communities, poses an additional risk. It further jeopardises an already fragile healthcare system and increases the burden on the remaining workforce. However, a detailed assessment of these concerns is lacking. Therefore, this systematic review aims to identify and analyse the major difficulties encountered by female healthcare workers in Pakistan, focusing on socio-cultural and institutional factors. The research question guiding this review was "What specific challenges are faced by women working in the healthcare sector of Pakistan?"

## Review

Materials and methods

Inclusion and Exclusion Criteria

The PEO (Population, Exposure, Outcome) framework was employed to establish the eligibility criteria for this review, as this framework is appropriate for descriptive research questions examining barriers or experiential phenomena where no comparison group is required. The components were defined as follows: population (female healthcare workers in Pakistan), exposure (workplace discrimination, harassment, cultural impediments, familial constraints, work-life balance issues, and safety concerns), and outcome (career attrition, mental health struggles, reduced professional advancement, and underrepresentation in leadership). No comparison group was required.

The inclusion criteria comprised studies published in English that were either randomised controlled trials (RCTs), nonrandomised clinical trials, or observational studies including thesis/dissertation related to the topic. The population of interest included female healthcare workers, encompassing physicians, dentists, pharmacists, nurses, medical assistants, and lady health workers. The intervention aspect focused on studies examining workplace and socio-cultural challenges faced by these women. The outcomes of interest included reports of workplace discrimination, issues related to work-life balance, harassment, and barriers to career advancement. There was no specific timeframe for the studies, and only those conducted within the context of Pakistan were considered.

Conversely, studies were excluded based on several criteria. These included reviews, case reports, animal studies, abstracts, non-completed studies, studies without results, editorials, and commentaries. Additionally, studies that focused exclusively on male healthcare workers or mixed-gender populations without specific data about female healthcare workers were omitted. Studies that did not address workplace or socio-cultural challenges and those that failed to report on obstacles faced by female healthcare workers were also excluded. Furthermore, studies published in languages other than English, those conducted outside of Pakistan, and studies assessed to have a high risk of bias or poor methodological quality were not included.

Data Sources

A comprehensive literature search was conducted across multiple databases, including PubMed/Medical Literature Analysis and Retrieval System Online (MEDLINE), Cochrane Library, Europe PubMed Central (Europe PMC), ScienceDirect, Elton B. Stephens Company (EBSCO) Open Dissertations, and ClinicalTrials.gov. The search was initiated on November 22, 2024, and concluded on December 5, 2024.

Search Strategy

The search strategy involved a combination of relevant keywords and Medical Subject Headings (MeSH) terms. Key concepts included challenges, problems, obstacles, barriers, difficulties, hurdles, issues, impediments, workplace challenges, socio-cultural challenges, employment issues, job-related difficulties, social constraints, and cultural issues. Additionally, terms related to female healthcare workers, such as "Female Healthcare Worker*", "women in healthcare", "female doctors*", "Lady Health Workers*", "female healthcare providers*", and "female workforce*", were incorporated. The search also targeted studies specifically related to Pakistan or the Pakistani healthcare system (Table 1).

**Table 1:** Search strategy MEDLINE: Medical Literature Analysis and Retrieval System Online; Europe PMC: Europe PubMed Central; EBSCO: Elton B. Stephens Company

Database	Search strategy	Number of studies before/after filters	Filters used
PubMed/MEDLINE	("pakistan*"[All Fields] OR "Healthcare Pakistan"[All Fields] OR "Pakistani healthcare system"[All Fields] OR "pakistan*"[All Fields] OR "healthcare pakistan*"[All Fields] OR (("ministries"[All Fields] OR "ministry"[All Fields] OR "ministry"[All Fields]) AND ("health"[MeSH Terms] OR "health"[All Fields] OR "health"[All Fields] OR "healthful"[All Fields] OR "healthfulness"[All Fields] OR "healths"[All Fields]) AND ("pakistan"[MeSH Terms] OR "pakistan"[All Fields] OR "pakistan*"[All Fields])) OR "Pakistani healthcare system"[All Fields] OR (("health"[MeSH Terms] OR "health"[All Fields] OR "health s"[All Fields] OR "healthful"[All Fields] OR "healthfulness"[All Fields] OR "healths"[All Fields]) AND ("sector"[All Fields] OR "sector s"[All Fields] OR "sectoral"[All Fields] OR "sectors"[All Fields]) AND ("pakistan"[MeSH Terms] OR "pakistan"[All Fields] OR "pakistan*"[All Fields])) OR ("challenge*"[All Fields] AND ("pakistan"[MeSH Terms] OR "pakistan"[All Fields] OR "pakistan"[All Fields]))) AND ((("female healthcare worker*"[All Fields] OR "women in healthcare"[All Fields] OR "female doctors*"[All Fields] OR "lady health workers*"[All Fields] OR "female healthcare providers*"[All Fields] OR "female workforce*"[All Fields] OR "health profession"[All Fields] OR "femal"[All Fields] OR "female"[MeSH Terms] OR "female"[All Fields] OR "females"[All Fields] OR "female s"[All Fields] OR "females"[All Fields]) AND (("delivery of health care"[MeSH Terms] OR (("delivery"[All Fields] AND "health"[All Fields] AND "care"[All Fields]) OR "delivery of health care"[All Fields] OR "healthcare"[All Fields] OR "healthcare s"[All Fields] OR "healthcares"[All Fields])) AND "worker*"[All Fields] AND "OR"[All Fields] AND (("womans"[All Fields] OR "women"[MeSH Terms] OR "women"[All Fields] OR "woman"[All Fields] OR "women s"[All Fields] OR "womens"[All Fields]) AND ("delivery of health care"[MeSH Terms] OR ("delivery"[All Fields] AND "health"[All Fields] AND "care"[All Fields]) OR "delivery of health care"[All Fields] OR "healthcare"[All Fields] OR "healthcare s"[All Fields] OR "healthcares"[All Fields])) AND "OR"[All Fields] AND (("femal"[All Fields] OR "female"[MeSH Terms] OR "female"[All Fields] OR "females"[All Fields] OR "female s"[All Fields] OR "femals"[All Fields]) AND "doctors*"[All Fields])) AND "OR"[All Fields] AND "lady"[All Fields] AND ("nurses, community health"[MeSH Terms] OR ("nurses"[All Fields] AND "community"[All Fields] AND "health"[All Fields]) OR "community health nurses"[All Fields] OR (("health"[All Fields] AND "visitors"[All Fields]) OR "health visitors"[All Fields])) AND "OR"[All Fields] AND (("femal"[All Fields] OR "female"[MeSH Terms] OR "female"[All Fields] OR "females"[All Fields] OR "female s"[All Fields] OR "female"[All Fields]) AND "nurse*"[All Fields])) OR "lady health workers*"[All Fields] OR (("femal"[All Fields] OR "female"[MeSH Terms] OR "female"[All Fields] OR "females"[All Fields] OR "female s"[All Fields] OR "female"[All Fields]) AND "health medical"[All Fields] AND ("technician"[All Fields] OR "technician s"[All Fields] OR "technicians"[All Fields])) OR "female healthcare providers*"[All Fields] OR "female workforce*"[All Fields] OR "health profession"[All Fields]) AND ((("challenge"[All Fields] OR "challenged"[All Fields] OR "challenges"[All Fields] OR "challenging"[All Fields] OR ("problem"[All Fields] OR "problem s"[All Fields] OR "problems"[All Fields]) OR ("obstacle"[All Fields] OR "obstacles"[All Fields]) OR ("barrier"[All Fields] OR "barrier s"[All Fields] OR "barriers"[All Fields]) OR ("difficulties"[All Fields] OR "difficulty"[All Fields]) OR ("hurdle"[All Fields] OR "hurdles"[All Fields]) OR ("issue"[All Fields] OR "issue s"[All Fields] OR "issues"[All Fields]) OR ("impediment"[All Fields] OR "impediments"[All Fields]) OR "workplace challenges"[All Fields] OR "socio-cultural challenges"[All Fields] OR "employment issues"[All Fields] OR "job-related difficulties"[All Fields] OR "social barriers"[All Fields] OR "SOCIAL CONSTRAINTS"[All Fields] OR "cultural issues"[All Fields]) AND "problem*"[All Fields]) OR "obstacle"[All Fields] OR "obstacles"[All Fields] OR "barrier*"[All Fields] OR "difficult*"[All Fields] OR "hurdle*"[All Fields] OR "issue*"[All Fields] OR "issues"[All Fields] OR "impediment*"[All Fields] OR "workplace challenges"[All Fields] OR "socio-cultural challenges"[All Fields] OR "employment issue*"[All Fields] OR "job related difficult*"[All Fields] OR "social barriers"[All Fields] OR "SOCIAL CONSTRAINTS"[All Fields] OR "cultural issues"[All Fields])	61/8	Free full text, adaptive clinical trial, clinical study, clinical trial, controlled clinical trial, evaluation study, multicenter study, observational study, pragmatic clinical trial, randomised controlled trial, English, humans, female, exclude preprints
Cochrane Library (CENTRAL)	Date Run: 02/12/2024 05:07:50 #1 (Challenges OR problems OR Obstacles OR barriers OR difficulties OR hurdles OR issues OR impediments OR “workplace challenges” OR “socio-cultural challenges” OR “employment issues” OR “job-related difficulties” OR “social barriers” OR “SOCIAL CONSTRAINTS” OR “cultural issues”) 115425 #2 (“Female NEXT Healthcare Worker” OR “women NEXT in healthcare” OR “female doctor” OR “Lady Health Worker” OR “female NEXT healthcare providers” OR “female workforce” OR “ health NEXT profession”) 39 #3 ( Pakistan OR Pakistani OR “Healthcare Pakistan” OR “Pakistani healthcare system”) 8744 #4 #1 AND #2 AND #3 9	9/3	Trials, English
ScienceDirect	(Challenges OR Obstacles OR “workplace challenges”OR “socio-cultural challenges” OR “employment issues” OR “job-related difficulties” ) AND ( “women in healthcare” OR “Lady Health Workers” ) AND ( Pakistan )	210/111	Research articles, open access
Europe PMC	(Challenges OR problems OR Obstacles OR barriers OR difficulties OR hurdles OR issues OR impediments OR “workplace challenges”OR “socio-cultural challenges” OR “employment issues” OR “job-related difficulties” OR “social barriers” OR “SOCIAL CONSTRAINTS” OR “cultural issues”) AND (“Female Healthcare Worker*”OR “women in healthcare” OR “female doctors*” OR “Lady Health Workers*” OR “female healthcare providers*” OR “female workforce*” OR “ health profession”) AND ( Pakistan OR Pakistani OR “Healthcare Pakistan” OR “Pakistani healthcare system”)	1324/1031	Full text in Europe PMC, research articles
EBSCO Open Dissertations	(Challenges OR problems OR Obstacles OR barriers OR difficulties OR hurdles OR issues OR impediments OR “workplace challenges”OR “socio-cultural challenges” OR “employment issues” OR “job-related difficulties” OR “social barriers” OR “SOCIAL CONSTRAINTS” OR “cultural issues”) AND (“Female Healthcare Worker*”OR “women in healthcare” OR “female doctors*” OR “Lady Health Workers*” OR “female healthcare providers*” OR “female workforce*” OR “ health profession”) AND ( Pakistan OR Pakistani OR “Healthcare Pakistan” OR “Pakistani healthcare system”)	8/8	English dissertations
ClinicalTrials.gov	Challenges OR problems OR Obstacles OR barriers OR difficulties OR hurdles OR issues OR impediments OR “workplace challenges” OR “socio-cultural challenges” OR “employment issues” OR “job-related difficulties” OR “social barriers” OR “SOCIAL CONSTRAINTS” OR “cultural issues” AND Female Healthcare Worker*”OR “women in healthcare” OR “female doctors*” OR “Lady Health Workers*” OR “female healthcare providers*” OR “female workforce*” OR “ health profession	0	Completed studies, interventional, observational studies, studies with results

Screening

After concluding the initial search and collecting the final studies, the selected articles were transferred to the Rayyan systematic review software (Rayyan QCRI, version 1.5.0, Qatar Computing Research Institute, Doha, Qatar) for future screening [15]. This web-based application facilitated the organisation and management of the studies, allowing for efficient collaboration among reviewers with blinded screening capabilities.

The screening process was conducted by four independent reviewers who assessed the titles and abstracts of the identified studies for eligibility. MA and DT screened records from PubMed/MEDLINE and Cochrane Library, HR screened records from Europe PMC and ScienceDirect, and AKM screened records from EBSCO Open Dissertations and ClinicalTrials.gov. All screened records were uploaded to Rayyan for centralised management and blinded review. Following initial screening, full-text articles were assessed for eligibility independently by MA, DT, HR, and AKM. Discrepancies at any stage were resolved through discussion, and when consensus could not be reached, a third author (IAM), who served as the mentor, was consulted for the final decision.

**Figure 1:**
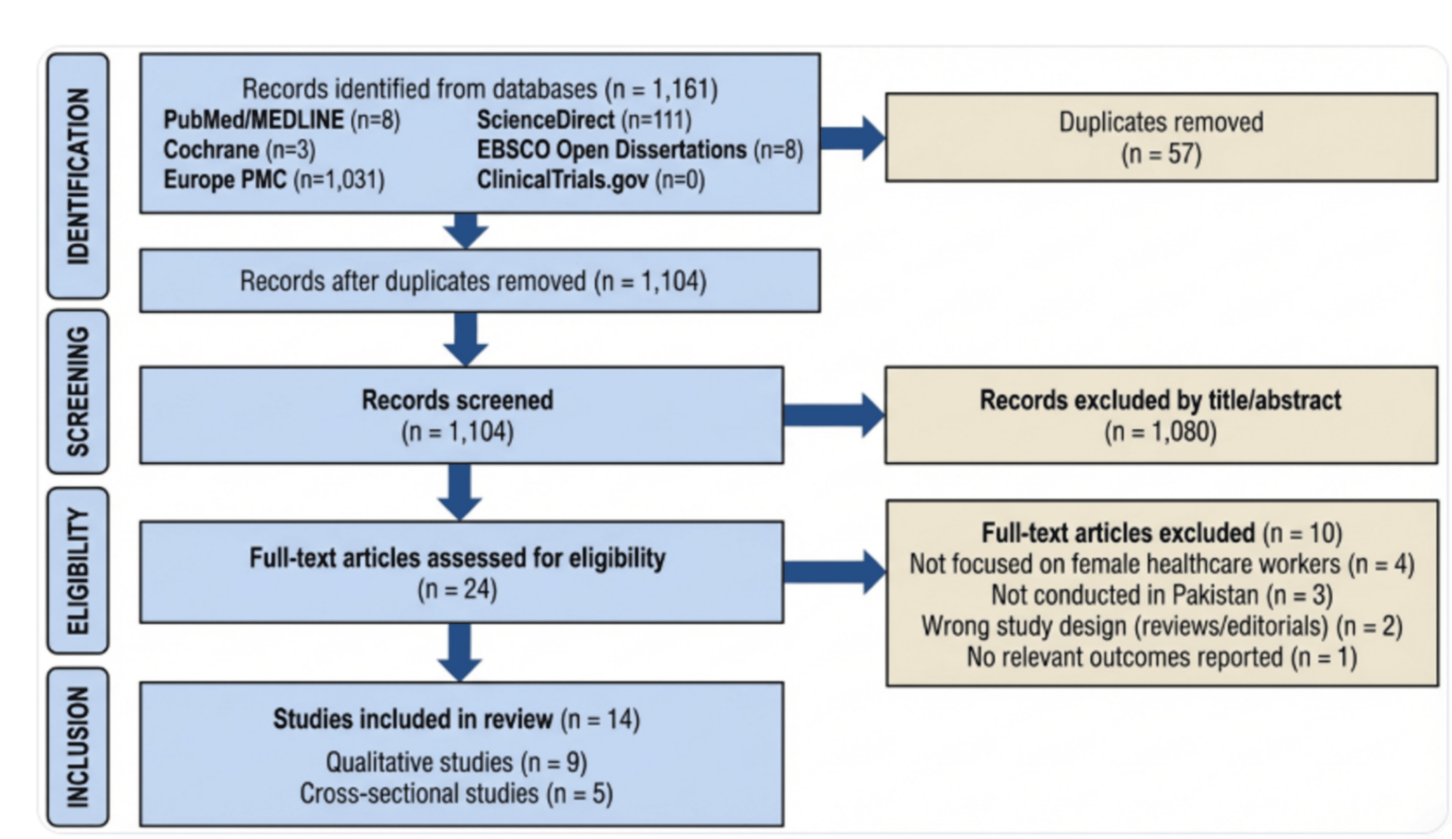
PRISMA flowchart PRISMA: Preferred Reporting Items for Systematic Reviews and Meta-Analyses; MEDLINE: Medical Literature Analysis and Retrieval System Online; Europe PMC: Europe PubMed Central; EBSCO: Elton B. Stephens Company The figure was created using BioRender.

Following the Preferred Reporting Items for Systematic Reviews and Meta-Analyses (PRISMA) 2020 guidelines [16], the initial search resulted in 1,161 records identified through database searching: PubMed/MEDLINE (n=8), Cochrane Library (n=3), Europe PMC (n=1,031), ScienceDirect (n=111), EBSCO Open Dissertations (n=8), and ClinicalTrials.gov (n=0). After removing 57 duplicates, 1,104 records were screened by title and abstract, resulting in the exclusion of 1,080 records. The remaining 24 full-text articles were assessed for eligibility, of which 10 were excluded (reasons: not focused on female healthcare workers, not conducted in Pakistan, inappropriate study design, or no relevant outcomes reported). Ultimately, 14 studies met the inclusion criteria, comprising nine qualitative studies and five cross-sectional studies, as illustrated in Figure 1.

Quality Appraisal of the Included Studies

Followed by careful screening, we identified a total of 14 studies, nine qualitative and five cross-sectional studies, for inclusion in our review. The Critical Appraisal Skills Programme (CASP) checklist was utilised for the assessment of the qualitative studies [17] by MA, DT, and HR and the Joanna Briggs Institute (JBI) critical appraisal checklist for analytical cross-sectional studies [18] by MA, AKM, and SM. This rigorous approach ensured that only those studies meeting acceptable quality standards were included in the final analysis. Disagreements in quality assessment were resolved through discussion, involving IAM when necessary.

The quality appraisal results, summarised in Table 2 and Table 3, indicate that the majority demonstrate good quality, with clear methodologies and robust data collection processes. This assessment underscores the reliability of the findings and their contribution to the existing literature on the subject.

**Table 2:** Quality appraisal of the included qualitative studies using the CASP checklist for the assessment of the qualitative studies Q1: Is there a clear statement of the aims of the research? Q2: Is the qualitative methodology appropriate for addressing the research aims? Q3: Is the research design appropriate to address the aims of the research? Q4: Was the recruitment strategy appropriate to the aims of the research? Q5: Were the data collected in a way that addressed the research issue? Q6: Has the relationship between the researcher and participants been adequately considered? Q7: Have ethical issues been taken into consideration? Q8: Was the data analysis sufficiently rigorous? Q9: Are the findings valid, and do they reflect the participants' views? Q10: How valuable is the research in terms of contributing to knowledge and understanding in the field? CASP: Critical Appraisal Skills Programme

Study	Q1	Q2	Q3	Q4	Q5	Q6	Q7	Q8	Q9	Q10
Raza et al. (2023) [19]	Yes	Yes	Yes	Yes	Yes	Yes	Yes	Partial	Yes	Yes
Iftikhar et al. (2023) [20]	Yes	Yes	Yes	Yes	Yes	Yes	Yes	Partial	Yes	Yes
Malik et al. (2023) [21]	Yes	Yes	Yes	Yes	Yes	Yes	Yes	Partial	Yes	Yes
Naqvi et al. (2023) [22]	Yes	Yes	Yes	Yes	Yes	Yes	Yes	Yes	Yes	Yes
Sarwar and Imran (2019) [23]	Yes	Yes	Yes	Yes	Yes	Yes	Yes	Yes	Yes	Yes
Khowaja et al. (2022) [24]	Yes	Yes	Yes	Yes	Yes	Yes	Yes	Partial	Yes	Yes
Ahmed et al. (2017) [25]	Yes	Yes	Yes	Yes	Yes	Yes	Yes	Partial	Yes	Yes
Chaudhry (2019) [26]	Yes	Yes	Yes	Yes	Yes	Yes	Yes	Yes	Yes	Yes
Nadeem and Yasmeen (2023) [27]	Yes	Yes	Yes	Yes	Yes	Yes	Yes	Yes	Yes	Yes

**Table 3:** Quality appraisal of the included cross-sectional studies using the JBI critical appraisal checklist for analytical cross-sectional studies Q1: Were the criteria for inclusion in the sample clearly defined? Q2: Were the study subjects and the setting described in detail? Q3: Was the exposure measured in a valid and reliable way? Q4: Were objective, standard criteria used for measurement of the condition? Q5: Were confounding factors identified? Q6: Were strategies to deal with confounding factors stated? Q7: Were the outcomes measured in a valid and reliable way? Q8: Was appropriate statistical analysis used? JBI: Joanna Briggs Institute

Study	Q1	Q2	Q3	Q4	Q5	Q6	Q7	Q8
Kumar et al. (2023) [28]	Yes	Yes	Yes	Yes	Yes	Yes	Yes	Yes
Raza and Khan (2023) [29]	Yes	Yes	Yes	Yes	Yes	Yes	Yes	Yes
Khan et al. (2012) [30]	Yes	Yes	Yes	Yes	Yes	Yes	Yes	Yes
Janjua et al. (2020) [31]	Yes	Yes	Yes	Yes	Yes	Yes	Yes	Yes
Ismail et al. (2022) [32]	Yes	Yes	Yes	Yes	Yes	Yes	Yes	Yes

Data Synthesis

Data extraction was performed independently by MA, DT, HR, and AKM using a standardised data extraction form developed by the study team. The extracted information included the following: author(s), year of publication, study design, study setting, sample size, participant characteristics (age, profession), key challenges identified, and recommendations proposed. Extracted data were verified for accuracy by SM.

Characteristics of the Selected Studies

The studies including a diverse population of female healthcare workers in Pakistan, such as physicians, surgeons, anaesthesiologists, dentists, pharmacists, nurses, midwives, lady health supervisors, lady health visitors, medical assistants, and female academicians in healthcare, were considered for this systematic review. The studies were conducted across various settings, including hospitals, academic institutions, and community health programs. The included 14 studies exploring the familial, professional, and socio-cultural barriers along with possible reported solutions are summarised in Table 4.

**Table 4:** Characteristics of the included studies IDI: in-depth interview; FGD: focus group discussion; KII: key informant interview; OR: operating room; JCPSP: Journal of College of Physicians and Surgeons Pakistan; MNCH: maternal, neonatal, and child health; F: female; exp: experience

Author (year)	Study design	Population	Sample size	Key challenges	Recommendations
Physicians and surgeons					
Raza et al. (2023) [19]	Qualitative (IDIs)	Non-practicing women doctors	59	Ineffective recruitment, harassment, patriarchal norms, family restrictions	Anti-harassment policies, flexible hours, childcare support
Iftikhar et al. (2023) [20]	Qualitative (IDIs)	Women in healthcare leadership	16	Gender bias, male insecurities, cultural influences	Mentorship programs, gender-responsive training
Naqvi et al. (2023) [22]	Qualitative (IDIs)	Female surgeons	9	Work-life imbalance, lack of mentorship, discrimination	Support networks, flexible training pathways
Nadeem and Yasmeen (2023) [27]	Qualitative	Female doctors (10-15 years exp)	9	Glass ceiling, discrimination, lack of mentors	Mentorship, leadership workshops
Janjua et al. (2020) [31]	Cross-sectional	Surgeons	47 F (98 total)	Gender discrimination in mentorship (80.6%) and OR opportunities (77.4%)	Awareness, support networks
Raza and Khan (2023) [29]	Cross-sectional	Female anaesthesiologists	17	Cultural stereotypes, work-family conflict, safety concerns	Part-time options, leadership training
Ismail et al. (2022) [32]	Cross-sectional	Authors in JCPSP	1,549 articles	Low female authorship (3.1% in anaesthesia)	Promote female representation
Academicians					
Malik et al. (2023) [21]	Qualitative (FGDs)	Healthcare academics	39	Lack of support, harassment, cultural issues	Daycare, transport, gender equity policies
Community health workers					
Khowaja et al. (2022) [24]	Qualitative (IDIs/FGDs)	Community midwives	30	Training gaps, acceptance issues, security concerns	Strengthen MNCH program, community engagement
Ahmed et al. (2017) [25]	Qualitative (IDIs)	Community midwives	20	Community resistance, family opposition, financial barriers	Community awareness, family engagement
Kumar et al. (2023) [28]	Cross-sectional	Community midwives	258	Financial difficulties, security, infrastructure issues	Salary improvement, transport, security
Khan et al. (2012) [30]	Cross-sectional	Lady health workers	97	Religious barriers (69%), socio-cultural (58%), transport (54%)	Awareness campaigns, incentives
Sarwar and Imran (2019) [23]	Qualitative	Healthcare professionals	25	Glass ceiling, stereotyping, organisational barriers	Organisational interventions, policy changes
Chaudhry (2019) [26]	Qualitative	Lady health workers	20	Professional recognition, work-life balance, safety	Policy reform, recognition programs

Challenges Identified

**Figure 2:**
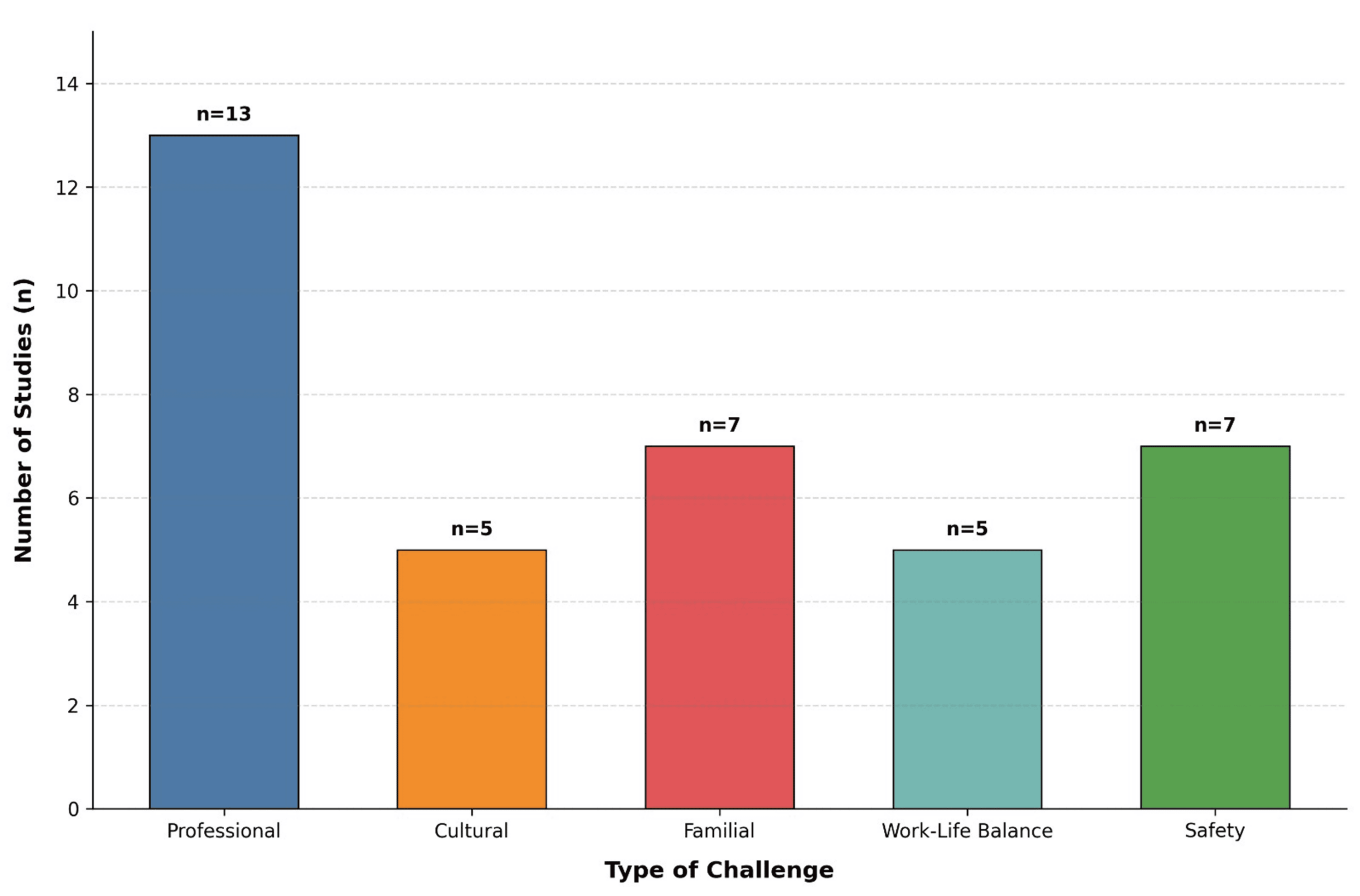
Challenges reported across different studies The figure was created using BioRender.

The 14 included studies reported challenges across five domains: professional aspects, cultural impediments, familial constraints, work-life balance, and safety concerns. The distribution of these challenges is presented in Figure 2. 

Professional Aspects

Professional obstructions were reported in 13 studies (92.9%) and included a lack of a robust merit-based recruitment system, improper placement in remote areas in the public sector, and a preference for experienced males in the private sector [19,21]. Low salaries compared to the workload, violence or harassment by patients and, sometimes, colleagues, and the lack of training programs, especially for community health workers, were of great concern [19,21].

Gender discrimination was also observed, including assignment of operating room procedures and representation in leadership and authorship positions [31,32]. Another significant factor is the low recognition of lady health workers in their roles in the workforce, which leaves them underpaid and lacking the necessary training [26].

Cultural Impediments

Cultural barriers were identified in five studies (35.7%). Patriarchal norms and expectations regarding preferences of marriage over career strictly at a certain age, Hijab/purdah restrictions, and the requirement to work in a female-only segregated environment were also limiting factors, especially in tribal areas [19-21,29,30].

Familial Constraints

Familial constraints were reported in seven studies (50%). Unfortunately, many women were not supported by their husbands or in-laws in working after marriage [19,24-27,29,30]. Child care is also considered a primary responsibility of females in Pakistani society, which restricts them from staying home and fulfilling household responsibilities [19,26,29].

Work-Life Balance

Work-life balance issues were documented in five studies (35.7%). Female healthcare workers struggled to manage dual professional and domestic responsibilities, with inflexible work schedules and a lack of institutional support exacerbating these challenges [19,20,22,26,29].

Safety Concerns

Safety concerns were reported in seven studies (50%). These included transportation difficulties, workplace insecurity, and fear of harassment or violence, particularly for community-based workers such as lady health workers and midwives operating in rural or conflict-affected areas [19,24-26,28-30].

Discussion

The findings of this systematic review paint a concerning portrait of a "leaky pipeline" within Pakistan's healthcare sector, where a powerful convergence of professional, cultural, and personal barriers diverts a significant portion of the skilled female workforce. Professional environments are often structurally inequitable, characterised not only by overt discrimination but also by implicit biases that marginalise women in leadership and academia [29,30,32]. A critical lack of formal mentorship and sponsorship, coupled with inflexible workplace policies and insufficient family support, forces many female healthcare workers to choose between career progression and familial obligations, contributing directly to high attrition rates [19,22,29].

Deep-seated socio-cultural norms profoundly reinforce these institutional failures. Unfortunately, the patriarchal structure of Pakistani society imposes a "double burden", where professional ambitions are systematically subordinated to prescribed gender roles as primary caregivers and homemakers [19,27]. Familial opposition, particularly from husbands and in-laws, remains a potent force restricting women's mobility and career continuity, a challenge acutely felt by community-based workers like lady health workers who also grapple with safety concerns and community skepticism [24,25,30]. This cultural context ensures that barriers are intersectional, meaning that women from lower socio-economic backgrounds or working in rural or conservative regions face compounded and often insurmountable obstacles.

Addressing this systemic crisis requires a multi-pronged and synergistic strategy. Policy-level interventions must include the stringent enforcement of anti-harassment laws and mandates for gender parity in leadership appointments. Concurrently, organisations must implement essential structural reforms, such as establishing formal mentorship programs, providing on-site childcare, an exclusive transport system for females only, and instituting flexible work schedules [20,22]. Crucially, these institutional efforts must be supported by community-level initiatives that engage religious and community leaders to shift societal perceptions and generate broader familial support for women's careers. Hence, to achieve health equity and strengthen the health imperative for Pakistan's population, female healthcare workers have to be empowered.

Comparison With Other Evidence

The findings of this systematic review align with and extend upon existing Pakistani evidence syntheses. Four recent reviews provide particularly relevant points of comparison.

Mahmood and Jan, synthesising 55 studies on the Lady Health Worker Programme efficiency, documented critical challenges including inadequate training and workload imbalance [33]. Critically, however, their review adopts a program-implementation lens, treating barriers as operational failures rather than gender-specific phenomena. By contrast, our synthesis reveals that identical barriers, that is, lack of mentorship, work-family conflict, and discrimination, affect physicians, nurses, and academics, suggesting that gender-based obstacles transcend professional boundaries. This cross-professional pattern is absent from Mahmood and Jan's analysis, likely because their exclusive focus on lady health workers obscures the systemic nature of gender discrimination across Pakistan's healthcare hierarchy.

Hashim et al. identified stress among lady health workers as stemming from overlapping workplace and household duties [34]. While their review conceptualises stress as a psychological outcome requiring individual-level coping strategies, our synthesis reframes this as a structural failure. The distinction matters: individual coping places responsibility on women to adapt; structural reform places responsibility on institutions to change. Hashim et al. do not critically examine why institutional supports (childcare, transport, flexible hours) remain absent despite decades of documentation, a silence that inadvertently legitimises the status quo.

Rehan et al. documented workplace violence against healthcare workers, finding verbal abuse most common and perpetrators typically patient attendants [35]. Our review corroborates this but identifies a notable divergence: Rehan et al. report no gender-disaggregated analysis of perpetration patterns, whereas our included studies (e.g., Raza et al.) suggest that harassment from male colleagues, not just patients, is a distinct and underreported category. This difference may reflect methodological choices: Rehan et al. included quantitative surveys that often collapse "colleague" and "patient" categories, while our qualitative studies allowed finer discrimination of perpetrator identity. Future reviews should examine whether colleague-perpetrated harassment carries different attrition risks than patient-perpetrated violence.

Qazi et al. introduced the "Doctor Brides" phenomenon, describing women who pursue medical education primarily for marital prospects [36]. While this framework is useful, it risks pathologising women's choices rather than criticising the structural constraints that produce them. Our review extends Qazi et al.'s work by documenting that family opposition continues throughout women's careers, spousal resistance to post-marriage work, in-law pressure to prioritise childcare, and restrictions on mobility, suggesting that the "Doctor Bride" framing is too narrow. We propose instead a "leaky pipeline across the life course" model, where barriers shift but never disappear: entry is negotiated through family permission, early career is constrained by childcare, mid-career is blocked by lack of mentorship, and leadership is foreclosed by glass ceilings.

A critical unresolved tension across all five reviews (including ours) is the near-absence of economic analysis. None of the included studies in any synthesis rigorously examine whether financial independence moderates attrition or whether women from higher socio-economic backgrounds experience barriers differently. This gap is striking because 50% of studies in our review reported familial constraints, yet none disaggregated by household income. Future research should test whether financial empowerment operates as a buffer against patriarchal opposition or whether cultural norms override economic considerations entirely.

Strengths and Limitations

Strengths:* *The studies considered for this systematic review have several strengths that enhance their credibility and relevance. Firstly, they encompass a diverse population of female healthcare workers in Pakistan, comprising doctors, nurses, and community health workers, providing a comprehensive understanding of the challenges faced across various professions. Additionally, many of these studies employed rigorous qualitative methodologies, such as in-depth interviews and focus group discussions, enabling rich, detailed data collection and a deeper exploration of participants' experiences. Furthermore, the majority of the studies demonstrated robust data collection processes and transparent methodologies, contributing to the reliability of their findings.

Our systematic review itself has several strengths that complement the included studies. The comprehensive search strategy utilised multiple databases, ensuring a broad range of literature was considered. The review adhered to the PRISMA 2020 guidelines [15], enhancing transparency and reproducibility in the research process. By synthesising findings from diverse studies, the evaluation effectively identified key themes, including workplace discrimination and cultural barriers, providing a holistic view of the challenges faced by female healthcare workers in Pakistan. The review also offers practical recommendations for interventions, such as mentorship programs and anti-harassment policies, to foster a supportive environment.

Limitations: The review has several limitations. First, although we searched within many databases, recognising that we focused solely on studies published in English may have left out important research published in Urdu or other local languages. This may have missed out significant viewpoints from community-based studies.

Second, the diversity in study designs (nine qualitative, five cross-sectional) and outcome measures hindered meta-analysis and restricted the direct comparability of the results across studies. Qualitative studies exhibited variability in their analytical methods (thematic versus content analysis) and the precision of theme derivation, as indicated in Table 2.

Third, publication bias is a concern because studies that report negative findings or a lack of challenges may not be published as often. The underreporting of economic difficulties and insecure employment in the included studies indicates potential selective outcome reporting.

Fourth, most included studies had relatively small sample sizes and were conducted in specific regions (e.g., Punjab, Khyber Pakhtunkhwa), which may limit generalisability to other provinces or rural-urban settings.

Fifth, the cross-sectional nature of the five studies precludes causal inference regarding the relationship between identified barriers and career attrition.

Clinical Implications

These findings suggest an urgent need for systemic changes within the healthcare sector. Creating mentorship opportunities, increasing remuneration packages, providing flexible working hours, promoting security against physical and verbal threats, introducing female-only transport services, implementing a strict anti-harassment policy, providing daycare facilities in hospital vicinity, and creating a recruitment system which accommodates the preferences of female healthcare workers can significantly enhance the work environment for female healthcare workers.

Future Research Directions

Longitudinal studies should focus on the experiences of underrepresented female healthcare professionals, such as community health workers, midwives, and assistants, and on the effects of targeted interventions on their retention and job satisfaction. Furthermore, qualitative research could offer a deeper understanding of the socio-cultural processes at work, helping create customised interventions that address the specific difficulties Pakistani women face in healthcare across a range of circumstances.

Recommendations

Based on the findings of this systematic review, we propose five targeted recommendations directly tied to the barriers identified across the 14 included studies.

First, structured mentorship programs should be established to address the lack of professional guidance reported in 13 studies (92.9%), a barrier explicitly cited by female surgeons [22], early-career physicians [27], and medical academics [21]. Without such programs, women remain excluded from informal professional networks that facilitate career advancement.

Second, on-site childcare facilities must be provided within hospital and clinic premises to mitigate work-life balance issues documented in five studies (35.7%) and familial constraints reported in seven studies (50%). Multiple participants across multiple studies described childcare as women's "primary responsibility", forcing many to reduce clinical hours or leave practice entirely after marriage [19,26,29].

Third, female-only transport systems should be introduced to address safety concerns reported in half of the included studies (50%). Transportation difficulties were identified as a critical barrier for community midwives [28], lady health workers [30], and female anaesthesiologists [29], particularly those operating in rural or conflict-affected areas such as Khyber Pakhtunkhwa.

Fourth, flexible work schedules and part-time training pathways must be implemented to accommodate the dual professional and domestic responsibilities that female healthcare workers face. Work-life balance issues were documented in five studies (35.7%), with inflexible rosters and lack of institutional support repeatedly cited as reasons for early career attrition [19,20,22].

Fifth, existing anti-harassment policies require strict enforcement alongside anonymous reporting mechanisms. Harassment, both overt and subtle, was documented across professional and community settings in 13 studies (92.9%), ranging from verbal abuse by patients to discriminatory assignment of operating room opportunities [31] and gender bias in promotion practices [27]. Policy enforcement without accountability mechanisms has proven insufficient.

Finally, for community-based workers, lady health workers and midwives, who face compounded barriers from lower socio-economic backgrounds and remote placements [24,25], salary improvements, security provisions, and community engagement initiatives involving religious and local leaders are essential complements to the institutional reforms listed above.

## Conclusions

This systematic review confirms that female healthcare workers in Pakistan face a convergence of professional, cultural, and familial barriers that impede career progression and drive attrition. Despite comprising nearly half of registered doctors, over one-third remain unemployed, a loss Pakistan's under-resourced healthcare system cannot afford. Based on these findings, we recommend enforcement of anti-harassment policies, structured mentorship programs, on-site childcare, flexible work schedules, and female-only transport systems. These reforms are essential not only for gender equity but for strengthening Pakistan's healthcare workforce and improving population health outcomes.
